# Dysphagia in children with repaired oesophageal atresia

**DOI:** 10.1007/s00431-016-2760-4

**Published:** 2016-08-20

**Authors:** Catelijne H. Coppens, Lenie van den Engel-Hoek, Horst Scharbatke, Sandra A. F. de Groot, Jos. M.T. Draaisma

**Affiliations:** 1 Department of Paediatrics, Radboudumc Amalia Children’s Hospital, PO box 9101, 6500 HB Nijmegen, The Netherlands; 2 Department of Rehabilitation, Donders Institute for Brain, Cognition and Behaviour, Radboudumc, Geert Grooteplein 10, 6525 GA Nijmegen, The Netherlands; 3 Department of Paediatric Surgery, Radboudumc Amalia Children’s Hospital, PO box 9101, 6500 HB Nijmegen, The Netherlands

**Keywords:** Dysphagia, Functional Oral Intake Scale (FOIS), Oesophageal atresia (OA), Oropharyngeal dysphagia, Videofluoroscopic swallow study (VFSS)

## Abstract

Dysphagia is a common problem in children with repaired oesophageal atresia (OA). Abnormalities in the oropharyngeal and oesophageal phase have hardly been studied. The aims of this study were to assess the prevalence of dysphagia in children with repaired OA and to identify and differentiate oral and pharyngeal dysphagia based on videofluoroscopic swallow study (VFSS) findings in a limited number of children in this cohort. Medical records of 111 patients, born between January 1996 and July 2013 and treated at the Radboudumc Amalia Children’s Hospital, were retrospectively reviewed. The prevalence of dysphagia was determined by the objective and modified Functional Oral Intake Scale (FOIS) in four age groups. The first performed VFSS of 12 children was structurally assessed. The prevalence of dysphagia was 61 of 111 patients (55 %) in age group <1 year. In age group 1–4, 5–11 and 12–18 years, the prevalence of dysphagia decreased from 54 of 106 (51 %) patients to 11 of 64 (17 %) and 5 of 24 (21 %) patients. The 12 VFSS’s reviews revealed oral dysphagia in 36 % and pharyngeal dysphagia in 75 %.

*Conclusions*: This study highlights dysphagia as an important problem in different age groups of children with repaired OA. Furthermore, our study shows the presence of oropharyngeal dysphagia in this population. This study emphasizes the need to standardize the use of objective dysphagia scales, like the modified FOIS, to provide a careful follow-up of children with repaired OA.

**Table:** 

**What is Known:** • *Prevalence of dysphagia in children with repaired oesophageal atresia varies widely (ranges from 45 to 70 %) in literature.* • *Oral, pharyngeal and oesophageal dysphagia require different treatment approaches.*	
**What is New:** • *We determined dysphagia based on functional oral intake and provide an overview of change in dysphagia prevalence and severity over time in children with repaired OA.* • *Our study shows that dysphagia, including oropharyngeal dysphagia, is highly prevalent in young children with repaired OA and improves with time.*

## Introduction

Oesophageal atresia (OA) is a congenital malformation, characterized by an interruption in the continuity of the oesophagus [11, 22]. OA affects one in 2500 to 4500 live births and is fatal without surgical treatment. The prognosis is influenced by the occurrence of associated morbidities [11, 17, 21]. Other congenital malformations are present in more than 50 % of children with OA [17]. The co-occurrence of the most frequent congenital anomalies is named the VACTERL association (vertebral, anorectal, cardiac, tracheo-oesophageal, renal and limb defects) [11, 17, 22]. During the previous two decades, survival rates of children with OA have improved to a current rate of more than 90 % [12, 17, 21, 22]. Despite this improved survival, significant numbers of children with repaired OA still have to deal with short- and long-term morbidity. This has led to increased interest in the identification of morbidity in these patients [10–12, 21, 23].

Dysphagia is a common problem [12, 20, 23]. Due to the dysphagia, many children develop adaptive feeding behaviours [11, 20]. Dysphagia can occur in association with gastro-oesophageal reflux [5, 6]. Moreover, serious consequences of dysphagia like failure to thrive and aspiration may occur [11]. Two issues limit the up-to-date knowledge of dysphagia in this population. First, different definitions are used to describe dysphagia [10, 14, 20], which probably explains the wide variability in prevalence of 45 to 70 % [12, 13, 18, 23]. Second, dysphagia can occur in one or more phases of the swallowing process, respectively, the oral, pharyngeal and oesophageal phase. Abnormalities in different phases require different treatment approaches [1, 8]. The extent to which dysphagia occurs in the oropharyngeal swallowing phase remains unclear [9, 26].

A clear definition and an objective tool are essential to accurately report the prevalence and severity of dysphagia during follow-up. According to the American Speech-Language-Hearing Association (ASHA) (www.asha.org), paediatric dysphagia is defined as ‘difficulty with any step of the feeding process, from accepting foods and liquids into the mouth to the entry of food into the stomach and intestines’. Appropriate non-invasive evaluation of change in dysphagia and its severity over time can be obtained using the Functional Oral Intake Scale [4]. To our best knowledge, no studies have described change in prevalence and severity of dysphagia over time using this functional oral intake scale in children with repaired OA.

Concerning the different swallowing phases, it is important to identify the specific phase in which dysphagia occurs. The videofluoroscopic swallow study (VFSS) is generally accepted as the best investigation to objectively assess the oropharyngeal phase of the swallow function [1, 8]. So far, occurrence of dysphagia in the oropharyngeal phase of the swallow has hardly been studied in children with repaired OA [9, 26].

## Aims

Identification of the change in prevalence and severity of, in particular, oropharyngeal dysphagia over time will provide advanced insight and may improve follow-up and management of children with repaired OA. The first aim of our study was to assess the prevalence and severity of dysphagia based on the Functional Oral Intake Scale (FOIS) in different age groups of children with repaired OA. In addition, our aims were to subdivide this prevalence in oropharyngeal and oesophageal dysphagia and to determine if dysphagia was associated with gastro-oesophageal reflux disease (GORD). Secondary, this study aimed to identify oral and pharyngeal dysphagia based on VFSS findings in a limited number of children in this cohort.

## Material and methods

### Patient population and materials

A retrospective cohort study in patients with OA, born between January 1996 and July 2013, was performed at the Radboudumc Amalia Children’s Hospital, Nijmegen, the Netherlands. Patients with OA treated in this tertiary paediatric centre were identified using the OA registration list of the Paediatric Surgery Department and were included in the clinical cohort. Patients with any of the following criteria were excluded: death within the first 6 months of life, patients with a follow-up less than 6 months, patients lost to follow-up or no available paediatric and paediatric surgery medical records. The medical records were reviewed from birth through December 2014.

Additionally, all first VFSSs of the included patients with repaired OA performed at the research location were identified. All included VFSSs were performed between June 2002 and November 2014. Patients included in the VFSS review will be referred to as ‘VFSS cohort’.

### Patient characteristics

Medical records of included patients in the clinical and VFSS cohort were reviewed for the following patient characteristics: data on gender, birth weight, gestational age, type of OA based on the Gross classification [7], associated malformations/syndromes, type of surgery to correct OA, oesophageal dilatation for anastomotic stricture, the reported presence of GORD and fundoplication. Our patients received standard anti-acid medication until the age of 6 months. Therefore, GORD was defined as use of anti-acid medication because of reflux symptoms after 6 months of age.

### Data collection

Medical records of patients included in the clinical cohort were systematically reviewed. The occurrence of dysphagia, sensations of food impaction, oesophageal dilatation and GORD was determined in four age groups: respectively <1, 1–4, 5–11 and 12–18 years. Patients were assigned to the age groups from birth until the age at last follow-up as stated in their medical record. The ordination of these age groups was based on anatomic differences of the swallowing mechanism in infants and adults [2], clinical experience of the paediatrician (JD) and two speech language pathologists (LE, SG) involved in this study.

### Dysphagia

To determine the prevalence and severity of dysphagia, the FOIS was used. This objective dysphagia scale was originally validated to determine change in the occurrence and severity of dysphagia in an adult population over time [4]. The FOIS was chosen since no appropriate functional oral intake tool exists to estimate change in dysphagia occurrence and severity over time in children [3]. The FOIS includes seven levels concerning functional oral intake, ranging from nothing by mouth (level 1) to total oral diet with no restrictions (level 7) as shown in Table 1 [4]. Based on information on oral intake as stated in the medical records, each patient was assigned to one of the FOIS levels in the four age groups. If no information concerning diet was stated, the oral diet was considered normal and rated as oral diet with no restrictions (level 7). If multiple levels were applicable within one age group, the lowest level was assigned.

**Table 1:** Functional Oral Intake Scale (FOIS) according to Crary et al. [4]: children 1–18 years

	Intake
Level 1	Nothing by mouth
Level 2	Tube dependent with minimal attempts of food or liquids
Level 3	Tube dependent with consistent oral intake of food or liquids
Level 4	Total oral diet of a single consistency
Level 5	Total oral diet with multiple consistencies, but requiring special preparations or compensations
Level 6	Total oral diet with multiple consistencies without special preparation, but with specific food limitations
Level 7	Total oral diet with no restrictions

Modifications in the FOIS were made in order to assign this scale to patients in age group <1 year, since infants at this age are still expanding their oral diet from liquid (milk) to pureed and solid foods. These modifications were made based on Christiaanse et al. [3] and based on the distinct stages in the process of expanding the diet in infants [16]. Normal expansion of oral diet was considered reached when introduction of solid foods in pureed form started before 9 months of age and the introduction of mashed foods and soft lumps started before 12 months of age. According to age group <1 year, the following FOIS modifications were made: level 4–level 6 were merged and assigned if expansion of oral diet was not reached. Level 7 was assigned if expansion of oral diet was reached (Table 2).

**Table 2:** Modified Functional Oral Intake Scale (FOIS): children <1 year

	Intake
Level 1	Nothing by mouth
Level 2	Tube dependent with minimal attempts of food or liquids
Level 3	Tube dependent with consistent oral intake of food or liquids
Levels 4–6	Expansion of oral diet not reached^a^
Level 7	Expansion of oral diet reached^a^

^a^Normal expansion of oral diet was considered reached when introduction of solid foods in pureed form started before 9 months of age and the introduction of mashed foods and soft lumps started before 12 months of age [16]

Dysphagia was defined as a FOIS level below 7; meaning total oral diet had specific food limitations or was more restricted. The severity of dysphagia was expressed in FOIS level (level 1–level 6). The occurrence of dysphagia was subdivided in oropharyngeal and oesophageal dysphagia. Therefore, sensations of food impaction and oesophageal dilatation in history were determined in the same age group. Dysphagia was considered to occur in the oesophageal phase if sensations of food impaction or oesophageal dilatation were reported**.**

### Videofluoroscopic swallow studies

Since the FOIS does not determine the aetiology of dysphagia, the prevalence of oral and pharyngeal dysphagia based on VFSS will give advanced insight in different possible causes of dysphagia in our population. The VFSS images were stored on video home system (VHS) or on the Digital Swallowing Workstation (Kay Pentax Swallowing Workstation, Lincoln Park, New Jersey). VFSS procedures in children are individualized according to the child’s age and developmental level. VFSS were completed with different volumes and three different nutritional consistencies if applicable for the patient, namely thin liquid (i.e. milk), liquid (i.e. pureed food) and solid food (i.e. bread). Contrast (Xenetic 300 mg or Barium; Guerbet, Brussels, Belgium) was used to visualize the swallow act [8, 24].

### Assessment procedure

The VFSS images were assessed according to structural and functional findings in the oral, pharyngeal and upper oesophageal phase of the swallowing process according to van den Engel-Hoek et al. [24]. The presence or absence of these findings was scored dichotomously.

As literature shows experience and training influences reliability, the following procedure was conducted in order to achieve accurate assessment of VFSS images [15]. First, two experienced speech language pathologists (LE, SG) assessed the VFSS images in real time and slow motion separately. The ratings were compared, and inconsistencies between the two raters were determined. These inconsistent VFSS ratings were reviewed and discussed until consensus was reached. In order to present the most reliable results, consensus was used to rate the identified abnormalities in the oral, pharyngeal and upper oesophageal phase.

### Statistical analyses

Data were analysed using SPSS statistics 20.0. Descriptive statistics were used for patients’ characteristics, prevalence and severity of dysphagia and VFSS findings. Categorical variables were described as number and percentage; continuous variables were described as median and interquartile range (IQR). The prevalence of dysphagia in patients with repaired OA was calculated in the four age groups. Additionally, the prevalence rate was subdivided in percentages oropharyngeal and oesophageal dysphagia. To provide a clear overview of changes in severity of dysphagia over time, severity was expressed by compromising the 7 FOIS levels into three categories, namely tube-dependent feeding (levels 1–3), oral diet with restrictions (levels 4–6) and oral diet without restrictions (level 7 = no dysphagia). Additional analyses were performed in order to compare patient’s characteristics and to determine if dysphagia was associated with GORD in the four age groups. For that, the two-tailed Fisher’s exact test was used with a significance level of 0.05. In order to compare change in dysphagia prevalence over time, a mixed logistic regression model with random intercept for subjects was used. This statistical model was chosen since each patient is present in multiple (>1) age groups and the variable of interest is on a dichotomous scale. A significance level of 0.05 was chosen.

## Results

### Patient characteristics

**Fig. 1:**
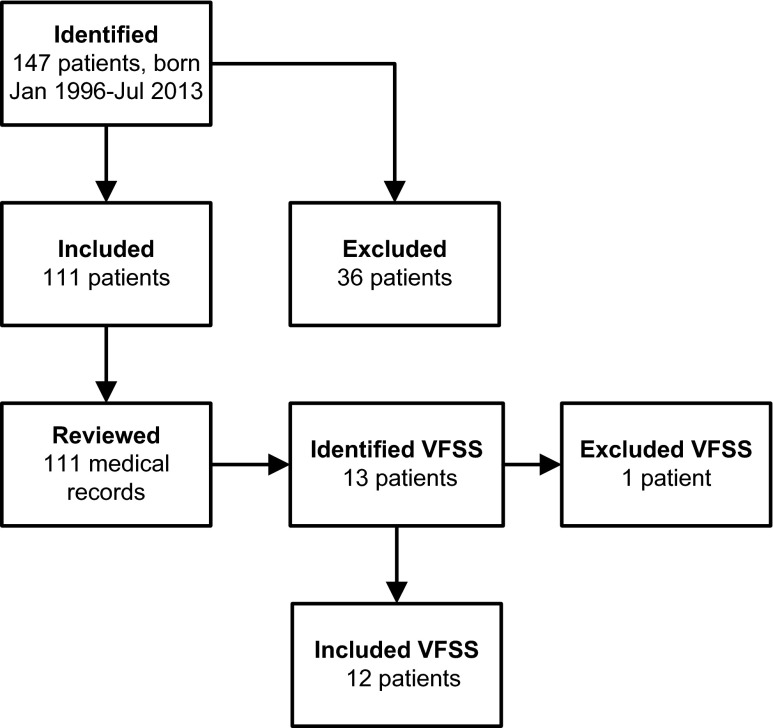
Selection of patients with repaired OA. *VFSS* videofluoroscopic swallow study

A total of 147 patients with repaired OA were identified. Of these, 36 patients were excluded: 13 patients died within the first 6 months of life, follow-up less than 6 months in nine patients, lost to follow-up in five patients and no available medical records in nine patients. So, 111 patients were included in the clinical cohort, as shown in Fig. 1. The number of patient records reviewed in the four age groups were as follows: 111 (<1 year), 106 (1–4 years), 64 (5–11 years), 24 (12–18 years). Characteristics of patients in the clinical cohort (*n* = 111) are shown in Table 3. Median patient age at last follow-up was 7.0 years (IQR, 2.9–11.4 years). Three patients died at, respectively, 7, 9 and 14 months of age.

**Table 3:** Characteristics of patients with repaired OA in the clinical and VFSS cohort

	Clinical cohort		Clinical cohort (excl. VFSS cohort)		VFSS cohort		
	*n* (%) or median (IQR)						*p* value^a^
Number of patients	111	(100 %)	99	(100 %)	12	(100 %)	
Age at last follow-up (years)	7	3–11	7	(3–11)	10	3–13	0.342
Age at VFSS performed (years)	2.2	1.3–4.9			2.2	1.3–4.9	
Gender							
Male	70	(63 %)	58	(59 %)	12	(100 %)	
Female	41	(37 %)	41	(41 %)			0.003
Gestational age (weeks)							
<37	37	(33 %)	33	(33 %)	4	(33%)	
≥37	68	(61 %)	61	(62 %)	7	(58%)	1.000
Unknown	6	(5 %)	5	(5 %)	1	(8%)	
Birth weight in grams^b^	2630	2135–3098	2640	2113–3123	2590	2435–3037	0,811
Associated syndromes							
No	69	(62 %)	61	(62 %)	8	(67 %)	
Yes	42	(38 %)	38	(38 %)	4	(33 %)	1.000
*VACTERL association*	30	(27 %)	27	(27 %)	3	(25 %)	
*Goldenhar syndrome*	2	(2 %)	2	(2 %)			
*Down syndrome*	3	(3 %)	2	(2 %)	1	(8 %)	
*Feingold syndrome*	1	(1 %)	1	(1 %)			
*Caudal duplication syndrome*	1	(1 %)	1	(1 %)			
*Unknown syndromes*	5	(5 %)	5	(5 %)			
Type of OA^c^							
Type C	86	(77 %)	76	(77 %)	10	(83 %)	
Other types of OA	23	(21 %)	21	(21 %)	2	(17 %)	1.000
*Type A*	9	(8 %)	8	(8 %)	1	(8 %)	
*Type B*	1	(1 %)	1	(1 %)			
*Type D*	6	(5 %)	6	(6 %)			
*Type E*	6	(5 %)	5	(5 %)	1	(8 %)	
*Other* ^d^	1	(1 %)	1	(1 %)			
Unknown	2	(2 %)	2	(2 %)			
Surgical procedure							
Primary anastomosis	90	(81 %)	81	(82 %)	9	(75 %)	
No primary anastomosis	17	(15 %)	14	(14 %)	3	(25 %)	0.401
*Delayed primary anastomosis*	1	(1 %)	1	(1 %)			
*Jejunum interposition*	8	(7 %)	7	(7 %)	1	(8 %)	
*Colonic interposition*	1	(1 %)	1	(1 %)			
*Ligation TOF*	5	(5 %)	4	(4 %)	1	(8 %)	
*Laser coagulation TOF*	1	(1 %)	1	(1 %)			
*Cervical oesophageal fistula* ^e^	1	(1 %)	0		1	(8 %)	
Unknown	4	(4 %)	4	(4%)			
Oesophageal dilatation							
Yes	79	(71 %)	71	(72 %)	8	(67 %)	
No	32	(29 %)	28	(28 %)	4	(33 %)	0.741
GORD^f^							
Yes	102	(92 %)	90	(91 %)	12	(100 %)	
No	9	(8 %)	9	(9 %)			0.593
Fundoplication							
Yes	16	(14 %)	13	(13 %)	3	(25 %)	
No	95	(86 %)	86	(87 %)	9	(75 %)	0.376

^a^ *p* value calculated for clinical cohort (excl VFSS cohort) vs VFSS cohort. ^b^Birth weight data were missing in 23 patients in the clinical cohort and in 5 patients in the VFSS cohort. ^c^Gross classification. ^d^OA type C with incomplete interruption of oesophageal lumen. ^e^Surgical procedure performed in foreign country. ^f^Overall GORD prevalence (age 0–18 years), prevalence per age group is shown in section: association between dysphagia and GORD

*OA* oesophageal atresia, *VFSS* videofluoroscopic swallow study, *IQR* interquartile range, *VACTERL* vertebral, anorectal, cardiac, tracheo-oesophageal, renal and limb malformations, *TOF* tracheo-oesophageal fistula, *GORD* gastro-oesophageal reflux disease

Of the clinical cohort, a total of 13 patients were identified with a VFSS procedure. One patient was excluded due to missing VFSS images. Eventually, a total of 12 VFSS, performed between June 2002 and November 2014, were included and assessed, as shown in Fig. 1. Patients’ characteristics of the VFSS cohort are shown in Table 3. In the VFSS cohort, all patients were male (*p* < 0.05). There were no other significant differences between patients’ characteristics in patients with (VFSS cohort) or without (clinical cohort excl. VFSS) a VFSS procedure.

### Dysphagia

The prevalence of dysphagia in the four age groups is shown in Table 4. Dysphagia was present in 61 (55 %) patients in age group <1 year and in 54 (51 %) patients in age group 1–4 years. In age group 5–11 years, the number of patients with dysphagia decreased (*p* = 0.001) to 11 (17 %). Dysphagia was present in 5 (21 %) patients in age group 12–18 years. The percentage oropharyngeal (30 %) and oesophageal dysphagia (70 %) remained stable.

**Table 4:** Prevalence of dysphagia, based on the Functional Oral Intake Scale, in children with repaired OA in age groups <1, 1–4, 5–11 and 12–18 years

Age group	<1 year		1–4 years		5–11 years		12–18 years	
Number of OA patients	111		106		64		24	
	% (*n*)	95 % CI	% (*n*)	95 % CI	% (*n*)	95 % CI	% (*n*)	95 % CI
Dysphagia	55 (61)	45–64	51 (54)^a^	41–61	17 (11)^b^	9–29	21 (5)^c^	7–42
Percentage oropharyngeal dysphagia^d^	37 (22)	25–50	21 (11)	11–34	27 (3)	6–60	20 (1)	1–71
Percentage oesophageal dysphagia^d^	63 (38)	50–75	79 (42)	66–89	73 (8)	39–94	80 (4)	28–99

^a–c^ *p* value was calculated to for change in percentage of dysphagia in ^a^ age group 1–4 compared to age group <1 year (*p* = 0,5126), ^b^age group 5–11 compared to age group 1–4 years (*p* = < 0,001), ^c^age group 12–18 compared to age group 5–11 (*p* = 0,8575). ^d^Data on sensation of food impaction and oesophageal dilatation in one patient with dysphagia were missing in age groups <1 and 1–4 years due to treatment in a foreign country until the age of 5 years

*OA* oesophageal atresia, *95 % CI* 95 % confidence interval for percentage was calculated

### Severity

**Fig. 2:**
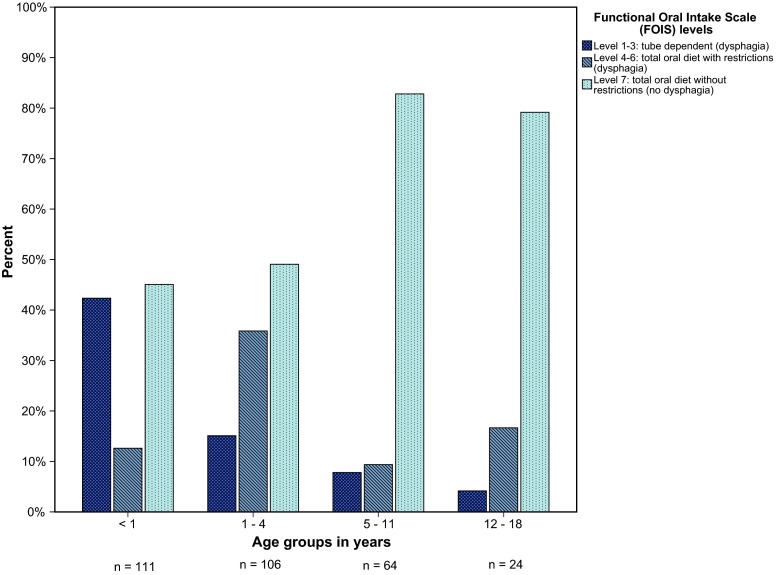
Severity of dysphagia, expressed in FOIS levels, in four age groups. *FOIS* Functional Oral Intake Scale, *n* number of patients per age group

The severity of dysphagia, expressed in FOIS levels, is shown in Fig. 2. The numbers of tube-dependent patients decreased in the older age groups from 47 (42 %) in age group <1 year to 16 (15 %) in age group 1–4 years, 5 (8 %) in age group 5–11 years and 1 (4 %) in age group 12–18 years. The number of patients with an oral diet with restrictions was 14 (13 %) in age group <1 year, 38 (36 %) in age group 1–4 years, 6 (10 %) in age group 5–11 years and 4 (17 %) in age group 12–18 years.

### Association between dysphagia and GORD

GORD prevalence was determined per age group. GORD was present in 100 (90 %) patients in age group <1 year, in 60 (57 %) patients in age group 1–4, in 28 (44 %) patients in age group 5–11 and in 8 (33 %) patients in age group 12–18 years. A statistically significant association was found with GORD and dysphagia in age group <1 year (*p* = 0.041), 1–4 years (*p* = 0.001) and 5–11 years (p = 0.001). GORD was more common in patients with dysphagia, (respectively, 97, 74 and 91 %) in the three age groups than that in patients without dysphagia (84, 40 and 34 %). There was no significant association (*p* = 0.289) in age group 12–18 years. Severe GORD, treated by fundoplication, was significantly associated (*p* < 0.05) with dysphagia in all four age groups.

### VFSS findings

A total of 12 patients were included in the VFSS review. Seven patients were tube dependent (FOIS levels 1–3), and 5 had a total oral diet with restrictions (FOIS levels 4–6) at the time of their VFSS assessment. Abnormalities in the different phases of the swallowing process are shown in Table 5. Oral dysphagia was present in four patients (36 %), and pharyngeal dysphagia was present in nine patients (75 %). Aspiration was identified in one patient. Abnormalities in the upper oesophageal phase were present in five (42 %) patients.

**Table 5:** Oral, pharyngeal and upper oesophageal abnormalities in the swallowing process based on VFSS findings

	*n*	(%)
Number of OA patients	12	(100 %)
Age at VFSS performed (years)		
<1	2	(17 %)
1–4	7	(58 %)
5–11	3	(25 %)
12–18	0	
Oral phase^a^	4	(36 %)
No bolus formation	2	(18 %)
Loss of food out of mouth	2	(18 %)
Piecemeal deglutition	1	(9 %)
Oral transport of liquid >3 s	0	
Pharyngeal phase	9	(75 %)
Material in valleculae or pyriform sinuses pre-initiation	9	(75 %)
Pharyngeal backflow	0	
Laryngeal penetration	0	
Aspiration	1	(8 %)
Post-swallow residue in valleculae	5	(42 %)
Post-swallow residue in pyriform sinuses or posterior pharyngeal wall or both	1	(8 %)
Upper oesophageal phase	5	(42 %)
Post-swallow residue on/in upper oesophageal sfincter	5	(42 %)

^a^Images of the oral phase in one patient were missing

*VFSS* videofluoroscopic swallow study

## Discussion

This retrospective study primarily assessed the prevalence of dysphagia, based on abnormal functional oral intake using the FOIS, in children (aged 0–18 years) with repaired OA. Prevalence of dysphagia was above 50 % in age groups <1 and 1–4 years. In age groups 5–11 and 12–18 years, prevalence rates decreased to approximately 20 %. The present study is the first reporting prevalence on dysphagia using the FOIS in children with repaired OA.

The prevalence of dysphagia in our study is consistent to other studies with regards to age groups <1 and 1–4 years [12, 13]. However, the prevalence of dysphagia in the age groups 5–11 and 12–18 years was lower than other literature reports, although our population was similar to those in previously published studies [12, 13, 18, 23]. In general, dysphagia in children is underreported as stated in literature [19]. Differences between our study and previously reported results might be explained by the use of various dysphagia definitions. In our study, we used the objective-modified FOIS. Obvious smaller differences were seen in prevalence rates compared to previous studies, if children with FOIS level 7 (no dysphagia) and sensations of bolus impaction or oesophageal dilatation in history were included in our prevalence analyses, respectively, 79 % in age group <1 year, 71 % in age group 1–4 years, 56 % in age group 5–11 years and 38 % in age group 12–18 years. This highlights the influence of dysphagia definitions on prevalence rates.

This study is, to our best knowledge, the only study in children with repaired OA grading severity of dysphagia using the objective-modified FOIS. Accurate determination of change in dysphagia severity is important to improve follow-up and evaluate treatment interventions [4]. Our results showed an overall decrease in prevalence and severity over the age groups. Application of an objective dysphagia scale to report change in dysphagia severity in OA patients might be a contributing factor to follow-up.

Our study confirmed the association of dysphagia with GORD, with or without fundoplication, in children with repaired OA. This is in accordance with previous research, which indicates abnormal oesophageal motility as common etiologic factor [5, 6]. Dysphagia and GORD can both cause aspiration, respectively, anterograde or retrograde aspiration [25]. The co-occurrence of dysphagia and GORD highlights the need to determine the aetiology of aspiration in children with repaired OA.

In our clinical cohort, dysphagia was subdivided into different swallowing phases. So far, this subdivision has not been applied before in dysphagia prevalence studies in children with OA [12, 13, 18, 23]. In our results, oropharyngeal dysphagia was present in children with repaired OA based on review of medical records. To objectively determine dysphagia in different swallowing phases, VFSS could be helpful [1, 8].

This study identified the aetiology of dysphagia based on VFSS findings in a limited number of patients. In our study, oral dysphagia was present in one third of our patients and pharyngeal dysphagia in more than three quarter of the patients in the VFSS cohort. Only Hörmann et al. [16] and Yalcin et al. [26] performed a VFSS study in children with repaired OA. First, differences in dysphagia prevalence should be considered in the light of a limited number of children in the studied cohorts. Hörmann et al. [9] published an article focusing on dysphagia in different swallowing phases using VFSS. In this study [9], all children had abnormalities in the pharyngeal phase, and none had abnormalities in the oral phase*.* Compared to this study, our percentage of oral dysphagia was higher, while pharyngeal dysphagia in our study was lower. A possible explanation for these results is the use of different consistencies in our study, whereby Hörmann et al. [9] only used thin liquid. Other consistencies can reveal additional abnormalities causing dysphagia [25].

Yalcin et al. [26] recently published a study to evaluate the functional disorders of deglutition in children with repaired EA with VFSS. They showed oral dysphagia in 10 % of the children with repaired esophageal atresia, suggesting that oral dysphagia may be associated with late onset of oral feeding. Our results support their hypothesis, since three of our four patients with oral dysphagia were tube dependent (FOIS levels 1–2 in age group <1 year). The percentage of pharyngeal dysphagia was more frequent in our population. This result may be explained by the fact that dysphagia was present in all our VFSS patients, while in Yalcin et al., dysphagia was absent in the majority of patients [26]. One of the issues that emerges from these findings is the correct indication of VFSS.

As a consequence of the retrospective study design, follow-up data were limited and therefore, not always straightforward. One researcher (CC) reviewed medical records and obtained data. However, in case of ambiguities, data were discussed with the involved paediatrician (JD) until consensus was reached. Concerning the VFSS findings, these do not reflect the overall OA population since selection of patients was based on the presence or absence of VFSS performed. Nevertheless, this study is the first to combine prevalence of dysphagia using the FOIS and identification of dysphagia in different swallowing phases using VFSS in children with repaired OA.

## Conclusion

Dysphagia prevalence in this study is consistent to other studies with regards to age groups <1 and 1–4 years and was lower in age groups 5–11 and 12–18 years. Our study showed that oropharyngeal dysphagia is present in children with repaired OA. This study emphasized the need to standardize the use of an objective dysphagia scale in follow-up of children with repaired OA. Using an objective dysphagia scale, like the modified FOIS, in children with repaired OA is necessary to give tailor made advices for feeding and swallowing in this patient group. Prospective studies using an objective dysphagia scale and VFSS in children with OA are warranted to correctly identify dysphagia in different swallowing phases.

## Abbreviations

ASHA: American Speech-Language-Hearing Association
FOIS: Functional Oral Intake Scale
GORD: Gastro-oesophageal reflux disease
IQR: Interquartile range
OA: Oesophageal atresia
TOF: Tracheo-oesophageal fistula
VACTERL: Vertebral, anorectal, cardiac, tracheo-oesophageal, renal and limb defects
VFSS: Videofluoroscopic swallow study
